# The Application of Adaptive Behaviour Models: A Systematic Review

**DOI:** 10.3390/bs8010011

**Published:** 2018-01-15

**Authors:** Jessica A. Price, Zoe A. Morris, Shane Costello

**Affiliations:** Faculty of Education, Krongold Clinic, Monash University, Learning and Teaching Building, 19 Ancora Imparo Way , Clayton, VIC 3800, Australia; zoe.morris@monash.edu (Z.A.M.); Shane.Costello@monash.edu (S.C.)

**Keywords:** adaptive behaviour, adaptive behaviour assessment, adaptive behaviour construct, systematic review, Vineland, ABAS

## Abstract

Adaptive behaviour has been viewed broadly as an individual’s ability to meet the standards of social responsibilities and independence; however, this definition has been a source of debate amongst researchers and clinicians. Based on the rich history and the importance of the construct of adaptive behaviour, the current study aimed to provide a comprehensive overview of the application of adaptive behaviour models to assessment tools, through a systematic review. A plethora of assessment measures for adaptive behaviour have been developed in order to adequately assess the construct; however, it appears that the only definition on which authors seem to agree is that adaptive behaviour is what adaptive behaviour scales measure. The importance of the construct for diagnosis, intervention and planning has been highlighted throughout the literature. It is recommended that researchers and clinicians critically review what measures of adaptive behaviour they are utilising and it is suggested that the definition and theory is revisited.

## 1. Introduction

Adaptive behaviour has been viewed broadly as “the effectiveness and degrees to which the individual meets the standards of personal independence and social responsibilities” [1] (p. 11). The construct includes skills that an individual requires in order to meet personal needs and to be able to cope with the social and natural demands in their environment. Specifically, Ditterline et al. (2008) noted that these skills involve being able to independently care for one’s personal health and safety, dress and bathe, communicate, behave in a socially acceptable manner, effectively engage in academic skills, recreation and work, and to engage in a community lifestyle [2]. However, there are a number of studies indicating that the construct of adaptive behaviour is still emerging and the definition had therefore been source of debate [3,4,5,6]. In particular, Harris and Greenspan (2016) argue that one of the key issues with the construct of adaptive behaviour is that it lacks an underlying theoretical framework that has never been fully resolved [7]. Therefore, the current review aims to explore the current definitions and applications of the models of adaptive behaviour to assessment tools within the literature.

The earliest reference to adaptive behaviour emerged in the 1800s during the Renaissance and Reformation period, as an adjunct to defining intellectual disability (ID) [8]. Despite the rising interest in adaptive behaviour, the introduction of intelligence testing decreased the popularity of the construct. In 1910, Binet and Simon related their assessment of the intelligence quotient (or IQ) to a table of traits distinguishing between “idiot”, “imbecile”, and “moron” [9]. Following this movement to assess ID in terms of IQ and social adaptability, Goddard revised the levels of mental retardation in terms of industrial capacity [9]. In 1920, Doll (1953) stressed the importance of utilising an ability assessment in diagnosing and treating individuals with ID [10]. Doll argued that assessments of individuals with ID were incomplete without valid estimates of adaptive behaviour, in which he termed social competence and social maturity at the time. In 1936, Doll developed what can be considered the first assessment of the adaptive behaviour construct, the Vineland Social Maturity Scale (VSMS), which aided the diagnosis of ID through a formal assessment of social competence and maturity [11]. The scale consisted of 117 items that measured an individual’s abilities and growth in relation to everyday situations, which consisted of three separate categories: self-help, locomotion and socialisation.

Building on this concept, the American Association on Intellectual and Developmental Disabilities (AAIDD), previously known as the American Association on Mental Retardation, formally included adaptive behaviour deficits as an integral part of the definition of ID in 1959. Their classification manual created a dual-criterion approach for the diagnosis of ID—intellectual functioning; and impairments in maturation, learning, and social adjustment. A review of the AAIDD’s manual took place in 1961, where the three initial impairments were altered to fall under an umbrella construct known as ‘adaptive behaviour’. The definition of adaptive behaviour comprised of two key elements:

1. The degree to which an individual is able to function and maintain him or herself independently; and
2. *The degree to which he or she satisfactorily meets the culturally imposed demand of personal and social responsibility* [12] (p. 61)

The origin of the modern conceptualisation of adaptive behaviour can be found in the diagnosis of ID. The Diagnostic and Statistical Manual of Mental Disorders—Fifth Edition (DSM-5) is a guide for the diagnosis of mental disorders [13]. It is imperative that a reliable diagnosis is made such that appropriate recommendations and treatments can be recommended. The construct of adaptive behaviour remains a vital component for the diagnosis of ID under diagnostic criteria B:

*“Deficits in adaptive functioning that results in failure to meet developmental and sociocultural standards for personal independence and social responsibility. Without ongoing support, the adaptive deficits limit functioning in one or more activities of daily life, such as communication, social participation, and independent living, across multiple environments, such as home, school, work, and community”*.

[13] (p. 33)

The International Classification of Diseases (ICD-10) is the diagnostic tool for “epidemiology, health management, and clinical purposes” [14]. Under Chapter V, classification code F70–F79, the ICD-10 includes the definition and criteria for a diagnosis of Mental Retardation (this term is under review for the ICD-11; due to be released 2018). The ICD-10 diagnosis bears similarities to that of the DSM-5; however, in terms of adaptive behaviour, the criteria clearly highlight a four-factor model: cognitive, language, motor, and social abilities. Specifically, the ICD-10 states that mental retardation is *“a condition of arrested or incomplete development of the mind, which is especially characterised by impairment of skills manifested during the developmental period, skills which contribute to the overall level of intelligence, i.e., cognitive, language, motor, and social abilities. Retardation can occur with or without any other mental or physical condition”* [14] (F70–F79).

Building on the AAIDD definition and the requirements for an adaptive behaviour measurement in the DSM and ICD, Nihira, Fosterm, Shellhaas and Leland (1968) were awarded funding to develop a tool that measured adaptive behaviour [15]. Nihira and his colleagues published the first standardised assessment instrument of adaptive behaviour, which they termed the Adaptive Behaviour Checklist (1968). This scale has since been revised twice and is now known as the AAMD Adaptive Behaviour Scale (1993). In 2000, Harrison and Oakland developed the Adaptive Behaviour Assessment System (ABAS) [16]. This assessment measure aligned with the three-factor structure outlined by the AAIDD. It was developed to assess the conceptual, social, and practical domains of adaptive functioning [17]. The original measure has since been reviewed and the third edition (2015) measures adaptive behaviour skills from birth to 89 years.

Similarly, the Vineland Adaptive Behaviour Scales (VABS) defined the construct of adaptive behaviour as a three-factor structure, including the broad domains of communication, daily living skills, and socialisation, but also has an optional measurement of motor skills (>7 years old and <50 years old), and maladaptive functioning [18]. The most recent version of the VABS is the Vineland-3, published in 2016. Sparrow, Cicchetti and Saulnier (2016), describe the Vineland-3 as a multipurpose tool, which can be used to support diagnoses, determine eligibility or qualification for special services, plan rehabilitation or intervention programs, and track and report progress [19].

Adaptive behaviour remains an emerging construct in terms of both conceptualisation and measurement [6]. The DSM-5 and the ICD-10 highlight the requirement of a measurement of adaptive behaviour for diagnostic purposes; however, there is a lack of clarity regarding the definition of adaptive behaviour. Jenkinson (1996) eloquently stated, “the only definition on which authors seem to agree is that adaptive behaviour is what adaptive behaviour scales measure” [20] (p. 99). Arguably this is an issue for most psychological constructs—being defined by how they are measured. However, as can be seen with the history of intelligence theory and assessment, adaptive behaviour as a construct requires more attention to develop to the same extent as intelligence. This point can be emphasised with the *Cattell–Horn–Carroll* (CHC) theory of intelligence, which is considered the most comprehensive and empirically supported psychometric theory of the structure of cognitive and academic abilities to date [21]. By the late twentieth century, the tests of the time did not reflect the diversity of the substantial evidence for the eight or nine broad cognitive Gf-Gc abilities (for example, the Wechsler Adult Intelligence Scale—Revised only measured two or three CHC abilities adequately). The CHC theory is the foundation on which many new and recently-revised intelligence batteries have been based [22].Therefore, as intelligence theory develops, the assessment tools that measure intelligence are revisited and reviewed. It is argued that the theory of adaptive behaviour has not undertaken the same level of attention and rigor as intelligence. Due to the extensive history and pivotal role of adaptive behaviour for diagnoses, this paper aims to provide a comprehensive overview of the application of adaptive behaviour models to assessment tools within the literature through systematic review.

## 2. Materials and Methods

### Literature Search

The methodology applied for systematically identifying relevant adaptive behaviour models was based on the terminology used throughout history and the common definitions noted in the introduction. The first database utilised in the review was *PsychINFO*, given that the database contains more than four million records and upwards of over 4000 expertly-indexed records added each week [23]. Subsequently, the database was searched using the terms summarised in Table 1 for works that contain one of the keywords or keyword combinations in their title, abstract, or list of keywords.

The language of the papers was limited to English. The year of publication was not limited in the search; however, only papers that appeared in peer-reviewed academic journals were considered relevant (i.e., book chapters and conference papers were excluded). All papers identified in the search process were checked for relevance by reading the article in full (the deduction process is demonstrated in Table 2). Papers were considered relevant for this review if the following criteria were met:

1. The central focus of the paper must be adaptive behaviour/functioning
2. The paper must discuss the model, theory or assessment tools that they have utilised for the study (e.g., whether they utilised the Vineland definition, or the Vineland scales for measurement of adaptive behaviour)

After the results were gathered and analysed from the initial search, a secondary search was conducted to analyse further assessment measures and tools of adaptive behaviour. Given the focus on psychological assessment measures, the secondary search utilised two databases: *Health and Psychosocial Instruments* (HPI), which provides information about behavioural measurement instruments, and *Mental Measurements Yearbook and Tests in Print* (MMYTP), which provides the most current descriptive test data. The search terms used in the secondary search were ‘adaptive behavior (with enabling multiple spellings of the word) or adaptive function * (with * = truncation), given that the narrow focus of the databases is already restricted to material containing psychological assessment measures.

## 3. Results

The results of the search and study selection are shown in Table 2. The original search process carried out in April–July 2017 produced 3974 articles, 107 of which were selected for full review. Of these, 75 studies did not meet the inclusion criteria, thus 32 studies were selected for review. The summary characteristics of the included studies are located in Table 3. The identified assessment tools and their factor structures are located in Table 4. The available psychometric properties of each assessment tool follow in Table 5 (the validity properties listed are adapted from Messick (1995) [24]).

### 3.1. Year of Publication

**Figure 1:**
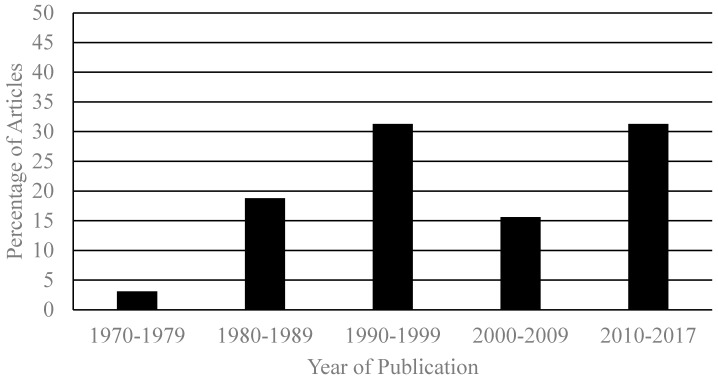
Percentage of reviewed articles published in each year.

The year of publication for the included articles ranged from 1972 to 2016. See Figure 1 for graphical representation.

### 3.2. Location

Twenty-five of the 32 studies identified were carried out in the US (78%), two studies were carried out in Spain and one study was conducted each in Turkey, Canada, Israel, the UK and Australia.

Eighty-four percent of the target populations identified within the search were clinical samples. Ten of the 32 studies had participants with known or suspected ID, and seven of the studies had participants with known or suspected Autism Spectrum Disorder (ASD). Of the remaining 15 studies, seven had normative samples and the remaining eight articles utilised clinical samples containing individuals with either developmental delay, cerebral palsy, William’s syndrome, hospital treatment for schizophrenia or depression, premature birth, learning and behavioural problems, high-risk populations and symptomatic HIV infection.

### 3.3. Adaptive Behaviour Assessment Tools

Twelve different adaptive behaviour assessment tools were identified within the review. While at face value some of the included scales (for example; PEDI-CAT, Coping Inventory and Role Functioning Scale) are not strictly measurements of adaptive behaviour, the studies have utilised these scales for this purpose and were therefore included [30,32,38]. The number of factors measuring adaptive behaviour ranged from two to six, with 33% highlighting a three-factor model. Table 4 below highlights the number of factors of each assessment measure and their associated subscales under overarching themes. The themes were determined through semantic and qualitative categorisation. The themes used within the table reflect the common definitions of the DSM-5: social, practical and conceptual; VABS: communication, socialisation, daily living skills; and AAIDD: social, practical and conceptual. Factors from each of the identified adaptive behaviour assessment scales were allocated to each of the themes based on qualitative categorisation. Furthermore, Table 5 highlights the psychometric properties available for the identified assessment scales. The results indicate that many of the aspects of construct validity are inadequate or not reported. The two most widely used assessment scales, ABAS-II and VABS-II, demonstrate the most psychometrically-sound properties.

The extensive history surrounding the emergence of adaptive behaviour highlights its importance for diagnoses and treatment planning. However, the term adaptive behaviour lacks clarity and a clear theoretical framework. The aim of this systematic review was to provide a narrative synthesis of the current frameworks and theoretical models within the literature. Searching across three electronic databases, 32 articles were identified which met the inclusion criteria. Despite the rigour of the review, it is difficult to provide conclusive results with regards to a common framework of adaptive behaviour. In fact, the current study further highlights the ambiguity and lack of agreement with regards to what the construct actually is.

Much of the research conducted on adaptive behaviour has focussed centrally on its measurement, rather than on theoretical aspects. The current study identified 12 different adaptive behaviour assessment tools that each measure the construct in alternative ways. The number of factors range from two to six depending on the assessment measure. A thematic analysis of each of the factors identified that all 12 assessment tools analysed social and practical components of adaptive behaviour; whereas, only half assessed communication or conceptual components. The two most prominent conceptual frameworks of adaptive behaviour (the AAIDD and the VABS) have some shared theoretical underpinnings, there are also clear differences; for example, the addition of motor skills and maladaptive behaviour domains within the VABS. Furthermore, there are clear differences amongst the reviewed assessment tools (albeit not equal contributors).This finding resonates with the same opinion of Jenkinson (1996) over 20 years ago, where he stated that the only definition of adaptive behaviour that authors agree upon is “that adaptive behaviour is what adaptive behaviour scales measure” [20] (p. 99). It is difficult to separate the construct of adaptive behaviour from the commercially-available adaptive behaviour scales. Therefore, it is apparent that the measurement of adaptive behaviour is driving policy and thereby becoming accepted as “theory”, rather than theory informing assessment. It is therefore important that the theoretical underpinnings of adaptive behaviour are challenged and explored further, given the widespread use and importance of adaptive behaviour as a diagnostic framework.

Furthermore, the articles analysed within this review indicate that many of the adaptive behaviour assessment scales available lack in many areas of construct validity. Despite having evidence for reliability and model-fit estimates, the six areas of construct validity identified by Messick (1995) are not adequately assessed nor have been made readily available [24]. According to a systematic review conducted by Floyd et al. (2017), the Vineland II scales demonstrate good content and external validity, adequate consequential validity and inadequate structural validity, in comparison to the ABAS-II scale, where evidence of content validity is considered good and structural and consequential validity are considered inadequate [54]. These findings highlight that even the most widely-used adaptive behaviour scales lack important aspects of construct validity. This indicates that it is difficult to determine the validity of research and assessment of current adaptive behaviour practices in Australia.

The results of the review suggested that although adaptive behaviour was of international interest, there was a strong western influence. Of the articles identified within this review, 78% were published by authors within the US. This finding suggests that the understanding of adaptive behaviour is shaped and influenced by the US. Furthermore, when taking into consideration that the definition of adaptive behaviour is primarily via assessment, a plausible conclusion is that adaptive behaviour is best understood as a reflection of typical behaviour within an American (or at least western) context, despite having clinical utility worldwide. Arguably this is problematic for many psychological constructs; however, the construct of intelligence has undertaken significant research to provide a sound theoretical underpinning and has been normed to apply to assessments cross-culturally. Some key assessments for adaptive behaviour have not been normed in different cultures, despite the cultural context required of an individual influencing what is considered ‘adaptive’. This finding resonates with the critical message highlighted by Jones (2010), where he suggested that psychology research is predominantly WEIRD—measures of western, educated, industrialised, rich and democratic cultures [56]. Jones cautioned that WEIRDos are not representative of other cultures and understanding human nature from a US perspective limits the generalisability of the research. When reflecting on this message and the findings from the current review, it can be argued that current research pertaining to the definition of adaptive behaviour may not be representative of all populations and consequently, requires a theoretical understanding from different cultural perspectives.

Specifically, within Australia, the measurement of the construct is required for diagnosis, practical applications and within legislative contexts. Given the importance of adaptive behaviour, it was unexpected that only one out of 32 articles had been published by an Australian author [20]. The study conducted by Jenkinson (1996) [20] aimed to explore the difficulties associated with the psychometric concepts and measurements to identify individuals with ID. After a review of the available assessment measures, he concluded that the introduction of adaptive behaviour measures has confounded the short-comings of standardised intelligence tests and that the construct needs a theoretical base in order to remain a psychological construct. Of the remaining articles, two were published in Spain and one article was published each in the United Kingdom, Israel, Canada and Turkey. Despite the studies being conducted in different countries, they all use existing western adaptive behaviour measurement tools to analyse adaptive behaviour. The findings of the current review highlight the need for a theoretical understanding of adaptive behaviour globally, and more specifically for different cultures and populations.

The data gathered within this review highlighted a pattern regarding the target populations used within the studies. Of the identified articles, 84.4% of the studies investigated clinical samples, with 21.8% focusing on individuals with ASD and 32.3% focusing on individuals with ID. Given that the diagnoses for ASD and ID require a measurement of adaptive behaviour to meet the criterion, this finding is not unexpected. However, it does stimulate suspicion of the validity of the construct of adaptive behaviour outside of these populations. The results indicated that 15.6% of the studies provided normative data for the ABAS. This highlights a particular strength of the ABAS and the paucity of normative data of comparative assessment tools. This review has highlighted that the construct of adaptive behaviour is basically defined by the measurements that assess the construct. The findings suggest that by continuing to focus on clinical samples, the construct of adaptive behaviour may be defined by the presentation of adaptive behaviour within these populations. The importance of investigating adaptive behaviour within clinical samples should not be undermined; however, the construct should also be defined within a typically-developing population.

## 4. Limitations

Given the nature of a systematic literature review, the current study is subject to its own intrinsic limitations. The current review may have been influenced by publication bias and time lag bias, where the identified articles may have been limited by studies that are accepted for publication and the rapid or delayed publication of research findings. In addition, the current study excluded studies that were not published in English. This exclusion criteria may have limited the number of identified studies published in other countries. However, given the review did return articles published in countries other than the US, but still utilised western adaptive behaviour assessment tools, it can be argued that studies published in other languages may follow the same westernised trend of adaptive behaviour.

## 5. Conclusions

The current study systematically reviewed the current frameworks and models of the construct of adaptive behaviour. The findings indicate that currently there is no universal definition of adaptive behaviour and that the definition is based predominantly on what the assessment tools measure. Specifically, the measurements are driving the theory, whereby US clinical populations are the central source of research. Whilst acknowledging the requirement of an adaptive behaviour measurement for funding access and diagnoses, it is advised that future researchers and clinicians critically review which definitions and measurements of adaptive behaviour they are utilising in their studies and practice, particularly given the lack of evidence for construct validity amongst scales. The major concern highlighted by the current review is that researchers are considering adaptive behaviour through one cultural lens, without having a robust theory underpinning its definition and understanding. This is problematic when the rating of adaptive behaviour may well depend on what the cultural context requires of an individual at different ages/stages. The possible consequences of taking such a narrow view of a critical construct are numerous, particularly given the strong reliance on adaptive behaviour in the DSM-5 and ICD-10 for diagnostic purposes. It is strongly recommended that the definition and theory of adaptive behaviour is revisited and increased evaluation is encouraged to ensure our research is current and valid to inform practice.

## Figures and Tables

**Table 1:** Syntax used in the systematic literature search for PsychINFO.

*PsychINFO* Search	
Keywords	Combined With
Adapt ^†^ behav ^†^	+ model ^†^
	+ theor ^†^
	+ structure
Adapt ^†^ function ^†^	+ model ^†^
	+ theor ^†^
	+ structure
Model ^†^	+ personal independence
	+ social responsibilit ^†^
	+ conceptual skill ^†^
	+ social skill ^†^ AND adapt ^†^ behav ^†^
	+ practical skill ^†^ AND adapt ^†^ behav ^†^

Note: ^†^= truncation of the word (a technique that broadens the search to include various word endings and spellings); + = and.

**Table 2:** Review protocol.

Filter Type	Descriptions and Guidelines	Results	
Inclusion Criteria	*Peer-reviewed journals:* Academic journal articles *Topic:* Articles with a focus on **models** and **theories** of **adaptive behaviour** *Language:* Limited to English *Time span:* No limitations		
Keyword Search	Search selected databases with the keywords defined in Table 1.	PsychINFO	HPI and MMYTP
	Initial hits:	3061	913
Deduction Process	*First deduction phase:* Ensure substantive relevance by requiring that all articles contain at least one keyword in their title, abstract or list of keywords. Exclusion criteria: Duplicate articles Articles written in another language Books Animal (i.e., rats) or artificial intelligence studies Obvious irrelevance to current topic		
	Reduced sample:	194	90
	*Second deduction phase:* Hits referencing solely adaptive behaviour measurement tools, rather than peer-reviewed articles were excluded		
	Reduced sample:	-	50
	*Third deduction phase:* Ensure relevance of content by subjecting all papers to a manual analysis of their abstracts		
	Reduced sample:	87	20
Content Analysis	Ensure relevance of content by requiring that the selected articles meet the criteria for inclusion (all articles read in full to examine their content and relevance)		
	Reduced sample:	21	11
**Final Sample**	Consolidation of initial and secondary search	**32**	

**Table 3:** Summary characteristics of included studies.

Author(s)	Year	Location	Sample	Target Population	Purpose	Design	Adaptive Behaviour Tool
Allen-Meares, P., & Lane, B. A. [25]	1983	United States	N/A	Special Education	Review of assessment tools for implications of practice for social workers	Review article	Adaptive Behaviour Inventory for Children (ABIC); Children’s Adaptive Behaviour Scale (CABS)
Arias, B., Verdugo, M. A., Navas, P., & Gomez, L. E. [26]	2013	Spain	*n* = 388 children aged 4–8 years old (61.5% male; 38.5% female)	Children with (*n* = 164) and without (*n* = 224) Intellectual Disability (ID)	Provide support to a multidimensional structure of conceptual, social and practical skills compared to a ‘*unidimensional*’ structure	Confirmatory Factor Analysis (CFA). The first steps were model specification and justification. Model fit was evaluated using a combination of absolute and incremental goodness of fit indices	Diagnostic Adaptive Behaviour Scale (DABS)
Aricak, O. T., & Oakland, T. [27]	2009	Turkey	*n* = 1690 children aged 5–21 (50% male; 50% female)	Representative of English-speaking U.S population as stratified by sex, race/ethnicity, and education levels consistent with the 1999 US census. Also included disorders proportionate to the population: ID, Asperger spectrum disorders, behaviour disorder, emotional disturbance, epilepsy, deaf and hard of hearing, speech impairment, and brain injury	To address the following four issues: whether (a) the skill areas in the ABAS-II-TRF, ages 5 to 21, display the same pattern of factor loadings, (b) the skill areas display the same factor loadings, (c) the intercepts of the observed skill areas are equal, and (d) the skill areas measure the corresponding factors with the same accuracy	Three two-group CFA were conducted to explore the factorial invariance of the ABAS-II-TRF. Comparative fit index (CFI), normed fit index (NFI), Tucker–Lewis index (TLI), root mean square error of approximation (RMSEA), and chi-square statistics were used as goodness-of-fit indexes to evaluate the nested models in this study	Adaptive Behaviour Assessment System-II Teacher Form (ABAS-II-TRF)
Bloom, A. S., & Zelko, F. A. J. [28]	1994	Canada	*n* = 117 children aged 9–111 months	Children with suspected developmental delay	To present data with respect to the variability in adaptive behaviour that may be expected in individuals who have varying degrees of intellectual delay	Chi-square comparisons and frequency comparisons	Developmental Profile II (DP-II)
Carpentieri, S., & Morgan, S. B. [29]	1996	United States	*n* = 40 *ASD* *n* = 20 (85% male; 15% female) *ID* *n* = 20 (60% male; 40% female)	Children with ID or ASD	To examine the relationship between adaptive functioning, as assessed by the VABS, and intellectual functioning, as assessed by the Stanford–Binet Intelligence Scale (SB), in children with ASD or ID. Second, the study aimed to determine how well predictions regarding level of functioning could be made from one measure to another	*T*-tests were applied to SB and VABS composite scores and MANOVA was applied to the four SB area scores and the three VABS domain scores to ascertain significant differences between the ASD and ID groups. Discriminant analyses were used to determine how accurately the SB area scores and VABS domain scores could classify the subjects into the two groups. Correlations between scores derived from the two measures were computed to determine significant relationships	VABS
Chiarello, L. A., Almasri, N., & Palisano, R. [30]	2009	United States	*n* = 319 children (58.9% male; 41.1% female) aged 3–13	Children with Cerebral Palsy (CP)	To describe the adaptive behaviour of children with CP and identify the child, family and service factors related to their adaptive behaviour	Descriptive analyses were computed for the children, family and service variables. Spearman and Pearson’s correlation coefficients were calculated to determine the strength and the direction of the relationships between scores for each child, family, and service variable and scores for the Adaptive Behaviour Index (ABI). Sequential regression analysis was conducted to examine the variance in the ABI. Post hoc analysis of whether children’s AB scores differed based on the extent of children’s communication and learning problems, and GMFCS levels were performed using a 3-way analysis of variance followed by the Bonferroni’s method for multiple comparisons.	The Coping Inventory
Childs, R. [31]	1982	United States	*n* = 50 (66% male; 34% female) aged 5–8	Elementary students with ID	To examine the adaptive behaviour of educable children with ID and its relationship with the diagnosis of ID as a two-dimensional concept	A 2 × 3 × 3 × 2 factorial ANOVA design incorporated the independent variables of race, sex, IQ, and placement, using 0.01 level of significance.	ABIC
Coster, W. J., Kramer, J. M., Tian, F., Dooley, M., Liljenquist, K., Kao, Y., & Ni, P. [32]	2015	United States	*n* = 365 parents of children with ASD aged 3–21	Children with ASD	To evaluate the performance of the PEDI-CAT with a national sample of children and youth with ASD	The structure and unidimensionality of the domains were evaluated using CFA and CFI and TLI were used to determine indexes of fit. The RMSEA was also examined. An IRT approach was used to obtain item parameter estimates for each of the domain item pools for youth with ASD. Logistic regression was used to examine differential item function (DIF)	The Pediatric Evaluation of Disability Inventory-Computer Adaptive Test (PEDI-CAT)
Duvdevany, I. [33]	2002	Israel	*n* = 64 *Study group* *n* = 33 aged 14–60 years old (27% male, 73% female) *Control group* *n* = 31 aged 14–60 years old (26% male, 74% female)	Study group—mild-moderate ID Control group—mild ID	To examine the effect of inclusion in the social activities of individuals with developmental disabilities in community centres in Israel on their perceived self-concept and their adaptive behaviour	Bivariate correlations among the self-control factors Hypothesis 1 was examined using a multiple analysis of covariance Hypothesis 2 was examined using several hierarchical multiple regression analyses. Further analysis was conducted with partial correlations between the self-concept factors and adaptive behaviour were performed.	ABS-Residential and Community 2nd edition
Fisher, M. H., Lense, M. D., & Dykens, E. M. [34]	2016	United States	*n* = 52 (52% male; 48% female) aged 14.2–48.9	Individuals with William’s Syndrome	To examine longitudinal profiles of cognitive and adaptive functioning in adolescents and adults with WS	A series of two-level mixed models were used to assess changes in KBIT-2 and VABS-II scores over time. Mixed models were used. Models were evaluated using restricted maximum likelihood estimation (REML)	Vineland Adaptive Behaviour Scale, 2nd Edition (VABS-II)
Fombonne, E., Siddons, F., Achard, S., Frith, U., & Happe, F. [35]	1994	United Kingdom	*n* = 19 (89% male; 11% female) aged 7.1–25.9 years	Children and adolescences with ASD	To explore the relationship between social cognition and everyday adaption in a French sample	Bivariate analyses and covariance analyses were used to statistically analyse the data	VABS
Freeman, B. J., Del’Homme, M., Guthrie, D., & Zhang, F. [36]	1999	United States	*n* = 440 children	Children with ASD	To examine how VABS scores in individuals with ASD change as a function of age utilising human growth modelling statistical technique	Mixed linear model (MLM) including both fixed and random components (e.g., fixed component—ANOVA; random component—random error)	VABS
Gillham, J. E., Carter, A. S., Volkmar, F. R., & Sparrow S. S. [37]	2000	United States	*N* = 95 ASD group *n* = 44 (81.8% male; 18.2% female) Pervasive Developmental Disorder Not Otherwise Specified (PDDNOS) *n* = 21 (85.7% male; 14.3% female) Developmentally Disordered group *n* = 30 (56.6% male; 43.4% female)	Children with either ASD, PDDNOS, or developmental disorder (mild-moderate ID *n* = 22, developmental language disorder *n* = 6, combination of motor skills and language delays *n* = 2)	To investigate the ability of the VABS to identify children with ASD	ANOVAs were conducted to determine whether there were significant differences between the three groups on IQ, mental age (MA) and VABS scores. MANOVA was conducted to examine group differences in VABS scores for the three adaptive domains and the two maladaptive scales. Pairwise Turkey tests were conducted. Discriminant function analysis was conducted to determine which measures of adaptive and maladaptive functioning from the VABS best predicted clinical diagnosis.	VABS
Goodman, S. H., Sewell, D. R., Cooley, E. L., & Leavitt, N. [38]	1993	United States	*n* = 112 women *n* = 79 study sample *n* = 33 control sample	Study sample—receiving outpatient treatment or had been hospitalized within the previous six months for either schizophrenia or severe depression Control sample—no history of psychiatric disturbance	To describe the RFS and provide reliability and validity data on one sample	Statistical analysis conducted for reliability (interitem reliability, test-retest reliability, and interrater reliability) and validity (criterion-group validity, and construct validity)	Role Functioning Scale (RFS)
Green, S, A., & Carter, A. S. [39]	2014	United States	*n* = 161 (79.5% male; 20.5% female) mean age = 28.2 months	Toddlers with ASD	To examine the development of daily living skills across 3 years in young children with ASD	Predictors of initial levels and trajectory of Daily Living Skills were examined by conducting a multilevel growth model analysis using hierarchical linear modelling (HLM). Correlations between daily living skills and parenting stress were examined at each time point to analyse the relationship. Subsequently, a hierarchical regression model was conducted to examine daily living skills as a predictor of parenting stress	VABS
Hogan, A. E., Scott, K. G., & Bauer, C. R. [40]	1992	United States	Mothers of *n* = 545 (50% male; 50% female) children, corrected age 36 months	Premature birth, with absence of severe health or neurological problems	The construction of a scale that was undertaken as an ancillary study to the larger Infant Health and Development Program (IHDP) 1990	Initial Scale Development—previously developed instruments were examined Extensive procedure for preliminary scale analyses including frequencies for item endorsement and exploratory factor analyses to determine the most appropriate number of scales	Adaptive Social Behavior Inventory (ASBI)
Huberty, T. J. [41]	1986	United States	*n* = 83 (51% male; 49% female) aged 7.5–16.2	Individuals referred for evaluation of learning or behaviour problems	To determine (1) the degree of relationship among the WISC-R and ABS-E factors and (2) whether the WISC-R deviation quotients (DQs) would be more accurate predictors of the ABS-SE scores than the IQ scores in an EMR sample	Separate stepwise regression analyses with maximum *R*^2^ improvements were conducted.	ABS-SE
Hunsucker, P. F., Nelson, R. O., & Clark, R. P. [42]	1986	United States	*n* = 1296 students from kindergarten through sixth grade (51% male; 49% female)	South-eastern school system—generally scores average or slightly above national norms on group achievement and IQ tests	Aimed to (a) revise the AAMD (now AAIDD) ABS-Public School Version by reducing its length and simplifying its scoring; (b) obtain local norms for the modified scale; and (c) to evaluate the modified scale using the criteria of reliability and validity	Detailed statistical processes described including: test-retest reliability, concurrent validity with Walker Problem Behaviour Checklist, discriminant validity between typically developing individuals and special education groups	ABS-Public School Version
Jenkinson, J. C. [20]	1996	Australia	N/A	Individuals with ID	To explore some of the difficulties inherent in the use of psychometric concepts and measurements to identify ID from a needs perspective	Review paper	N/A (AAIDD definition of AB discussed)
Kane, H., & Oakland, T. D. [43]	2015	United States	Archival data from the ABAS-II → *n* = 7370 persons from ages 0 through 89 stratified to be representative of the 1999 US census	Normative data from the ABAS-II	To investigate the nature of adaptive behaviour in high- and low-ability groups, with the specific intent of identifying whether patterns of differentiation exist	After forming high- and low-adaptive groups, a series of exploratory factor analyses compared the relative importance of the constructs measured by the ABAS-II across each ability group	ABAS-II
Keith, T. Z, Fehrmann, P. G., Harrison, P. L., & Pottebaum, S. M. [3]	1987	United States	*n* = 556 children aged 5–12	Children selected that overlapped with the standardization of the VABS and the Kaufman Assessment Battery for Children	To determine the nature of the relation between adaptive behaviour and intelligence	CFA was used to evaluate all three hypotheses within the study	VABS
Liss, M., Harel, B., Fein, D., Allen, D., Dunn, M., Feinstein, C., Morris, R., Waterhouse, L., & Rapin, I. [44]	2001	United States	*n* = 75 children High functioning ASD (HAD) *n* = 35 aged 84–113 months (77% male; 23% female) Low functioning ASD (LAD) *n* = 40 aged 107–113 months (85% male; 15% female) Developmental Language Disorder (DLD) *n* = 31 Low IQ *n* = 17	Children with ASD, DLD and low IQ	To investigate the relationship between adaptive behaviour, IQ, ASD symptomatology, and other cognitive skills in both HAD and LAD children and controls matched on both age and nonverbal IQ	Multiple correlations were utilised to analyse relationships To determine which cognitive and behavioural variables best predicted adaptive functioning, sequential regressions using SPSS were run for each adaptive behaviour domain for each group	VABS
Navas, P., Verdugo, M. A., Arias, B., & Gomez, L. E. [4]	2012	Spain	*n* = 388 children aged 4–8 years old (61.5% male; 38.5% female) → same sample as Arias et al. study	Children with (*n* = 164) and without (*n* = 224) Intellectual Disability (ID)	To present the develop of the AAIDD’s forthcoming DABS in Spain, specifically form 4–8 years old	The Partial Credit Model (PCM) was chosen to analyse items’ and persons’ functioning with the goal of selecting those items that will contribute to develop a short and precise measure of significant limitations in AB	DABS
Oakland, T., & Algina, J. [45]	2011	United States	*n* = 1350 children aged 0–5 years	The standardization sample of the ABAS-II—representative of the 2002 U.S census	To examine the factor structure of the ABAS-II parent/primary caregiver form for ages 0–5 years	Two-group CFA model were estimated using the robust maximum likelihood procedure (RMLP) Model comparison chi-square tests were conducted for use with the chi-square goodness-of-fit statistics produced by the RMLP	ABAS-II
Oakland, T., Illiescu, D., Chen, H., & Chen, H. [46]	2013	United States	*n* = 3131 *Romania*—*n* = 801 parents *Taiwan*—*n* = 660 parents *United States*—*n* = 1670 parents	All samples stratified by children’s age, gender, parent’s education level, and geographic region to be representative of each population	To examine possible similarities and differences in key psychometric qualities between the U.S-developed ABAS-II and its Romanian and Taiwanese adaptations	Reliability estimates were computed for the skill area and composite scores and then were compared across the three tests versions. Correlations between skill area and composite scores were computed and compared across the three versions to determine equivalence Results of CFA were also compared to determine factor structures	ABAS-II
Perozzi, J. A. [47]	1972	United States	N/A	N/A	Review paper with the focus on the three aspects of adaptive behaviour identified by the AAMD (now AAIDD)	Review	N/A (AAIDD definition of AB discussed)
Schatz, J., & Hamdan-Allen, G. [48]	1995	United States	*n* = 109 *ASD* *n* = 72 (80.5% male; 19.5% female) *ID* *n* = 37 (72.9% male; 27.1% female)	ASD and ID	Examination of the impact of age and intelligence upon the pattern of adaptive skills in children with ASD compared to those with ID	Statistical analyses utilised: - Chi-square goodness of fit - Hierarchical multiple regression - Bonferroni correction Power analysis	VABS
Seifer, R., Sameroff, A. J., & Jones, F. [49]	1981	United States	*n* = 1080 *Normative Sample* *n* = 323 mothers of 30-month-olds (50% male; 50% female)*n* = 309 mothers of 48-month-olds (55% male; 45% female) *High-Risk Sample* *n* = 234 mothers for 30-month-olds *n* = 214 mothers for 48-month-olds	Normative and High-risk population (high-risk = psychiatric diagnosis)	The present study had two functions: (1) to demonstrate that the RABI was an instrument which could yield maternal reports of specific areas of behavioural competence in preschool children; (2) to examine the difference in families with varying socioeconomic, racial, and psychiatric status	Interview of mothers using the RABI. Factor analytic procedures were used to reduce the interview data. Scales were created by summing the sores of high-loading items. Cronbach’s alpha statistics and corrected item-scale correlations were computed in the Normative samples	The Rochester Adaptive Behavior Inventory (RABI)
Stinnett, T. A., Fuqua, D. R., & Coombs, W. T. [5]	1999	United States	*n* = 3328 Children with ID (*n* = 2074), children without ID (*n* = 1254)	Children with and without ID	Examination of the construct validity of the ABS-S:2 through exploratory factor analyses	A series of factor analyses were performed on the correlation matrices reported in the ABS-S:2 manual	ABS-S:2
Tasse, M. J., Schalock, R. L., Balboni, G., Berasni, H., Borthwick-Duffy, S. A., Spreat, S., Thissen, D., Widamen, K. F., & Zhang, D. [6]	2012	United States	N/A	N/A	Aims to update the current conceptualization, measurement, and use of the adaptive behaviour construct	Critical review	ABS-S:2 ABAS-II VABS-II
Tasse, M. J., Schalock, R. L., Thissen, D., Balboni, G., Berasni, H., Borthwick-Duffy, S. A., Spreat, S., Widamen, K. F., Zhang, D., & Navas, P. [50]	2016	United States	*n* = 1532 *Phase 1* *n* = 474 individuals aged 4–21 (52% males, 48% females) *Phase 2* *n* = 1058 individuals aged from 4–21 (50% male, 50% female)	Representative U.S sample including race (73% Caucasian), ethnicity and known diagnoses	The development and standardization of the DABS	Detailed paper and statistical analysis on the development of the DABS within an American population and the standardization of the assessment tool	DABS
Wolters, P. L., Brouwers, P., Moss, H. A., & Pizzo, P. A. [51]	1992	United States	*n* = 25 children aged 1–12 years (68% male, 32% female)	Children with symptomatic HIV infection	The purpose of this study was to characterize how HIV disease affects the everyday behavioural functioning of children in their home environment, and to determine the therapeutic effect of 6 months of zidovudine therapy on adaptive behaviour.	ANOVA with split-plot design and Greenhouse–Geisser correction were used to evaluate pre to posttreatment performance for all subjects combined as well as by various subgroups. A hierarchical approach to the analysis was used to reduce the possibility of Type 1 errors	VABS

**Table 4:** Factors from adaptive behaviour assessment scales mapped across themes.

Scales	Factors	Communication	Social	Practical	Conceptual	Miscellaneous
RABI ^1^	6	Family communication	Social behaviour with peers	Behaviour around the home	Content of play	Other Caretakers Miscellaneous Behaviours
ABIC ^2^	6	-	Family Community Peer relations	Non-academic school roles Earner/consumer Self-maintenance	-	-
ABS-S ^3^	5	-	Social adjustment	Personal self-sufficiency Personal adjustment	Personal-social responsibility	Community self-sufficiency
CABS ^4^	5	Language development	Socialisation	Independent functioning Economic-vocational activity	Family role performance	-
DP-II ^5^	5	Communication	Social	Physical Self-help Academic	-	-
PEDI-CAT ^6^	4	-	Social/cognitive	Daily activities Mobility	Responsibility	-
RFS ^7^	4	-	Immediate social network relationships Extended social network relationships	Working productivity Independent living and self-care	-	-
ABAS-II ^8^	3	-	Social	Practical	Conceptual	-
ASBI ^9^	3	Disrupt	Express	Comply	-	-
DABS ^10^	3	-	Social	Practical	Conceptual	-
Vineland III ^11^	3 *2 optional*	Communication	Socialisation	Daily living skills Motor skills Maladaptive behaviour	-	-
CI ^12^	2	-	Environment	Self	-	-

Note: Superscript denotes scale authors: ^1^ = Jones (1977); ^2^ = Mercer (1979); ^3^ = Lambert, Nihira & Leland (1993); ^4^ = Richmond & Kicklighter (1980); ^5^ = Alpern, Boll & Shearer (1980); ^6^ = Haley, Coster, Dumas, Fragala-Pinkham & Moed (1992); ^7^ = not reported; ^8^ = Harrison & Oakland (2003); ^9^ = Hogan, Scott & Bauer (1992); ^10^ = AAIDD; ^11^ = Sparrow, Cicchetti & Saulnier (2016); ^12^ = Zeitlin (1985).

**Table 5:** Available psychometric properties for identified assessment scales.

Scales	Age Range	Model-Fit and Reliability	Content Validity	Substantive Validity	Structural Validity	Generalizability	External Validity	Consequential Validity
RABI [49]	2–6	Inter-rater reliability 0.84–0.99	N/A	N/A	N/A	N/A	N/A	N/A
		Intra-scale reliability 0.63						
ABIC [25,31]	5–11	Subtest reliability > 0.77 [52]	N/A	N/A	N/A	N/A	N/A	N/A
		Split-half > 0.96 [52]						
ABS-S [5,6,33,41,42]	3–18	No composite score	Considered adequate [53]	N/A	Considered adequate [53]	N/A	N/A	Considered inadequate [53]
		Test-retest reliability 0.90						
CABS [25]	5–10	N/A	N/A	N/A	N/A	N/A	N/A	N/A
DP-II [28]	Birth—7	N/A	N/A	N/A	N/A	N/A	Specificity 67% [54]	N/A
							Sensitivity 50% [54]	
PEDI-CAT [32]	Birth—20	Comparative Fit Index 0.93–0.98	N/A	N/A	N/A	N/A	Intra-class correlation > 0.95	N/A
RFS [38]	Not reported	Inter-item reliability 0.92	N/A	N/A	N/A	N/A	Prediction accuracy of 78.8% Correlations 0.28–0.59	N/A
		Test-retest reliability 0.68–0.92						
		Inter-rater reliability 0.21–0.82						
ABAS II [6,27,43,45,46]	Birth—89	Comparative Fit Index 0.96	Considered good [53]	N/A	Considered inadequate [53]	N/A	N/A	Considered inadequate [53]
		Internal consistency < 0.80						
		Test-retest reliability < 0.90						
		Inter-rater reliability < 0.60						
ASBI [40]	3 (utilised in study)	Scale reliabilities 0.71–0.79	N/A	N/A	N/A	N/A	N/A	N/A
DABS [4,26,50]	4–21	Comparative Fit Index < 0.95	N/A	N/A	Composite reliability 0.76–0.88	N/A	Specificity 89–91% [55]	N/A
							Sensitivity 81–98% [55]	
Vineland II [3,6,29,34,35,36,37,39,44,48,51]	Birth—90	Internal consistency < 0.90	Considered good [53]	N/A	Considered inadequate [53]	N/A	Considered good [53]	Considered adequate [53]
		Test-retest reliability < 0.80						
		Interrater reliability < 0.70						
CI [30]	3–16	Internal consistency 0.84–0.98	N/A	N/A	N/A	N/A	‘shows evidence of discriminant validity’ [30] (p. 437)	N/A
		Interrater reliability 0.78–0.92						

Note: References to articles are included in brackets; Despite having newer versions available for Vineland and ABAS scales, analysed articles referenced earlier versions.
